# A coalition-driven examination of organization capacity to address food insecurity in Greater Houston: a qualitative research study

**DOI:** 10.3389/fpubh.2023.1167100

**Published:** 2023-08-15

**Authors:** Jemima C. John, Jennifer Gonzalez, Sara-Grace Chan, Heidi McPherson, Jennifer N. Aiyer, Esperanza Galvan, Nicole Browning, Shreela V. Sharma

**Affiliations:** ^1^Department of Epidemiology, Human Genetics and Environmental Sciences, School of Public Health, The University of Texas Health Science Center at Houston, Houston, TX, United States; ^2^Harris Health System, Houston, TX, United States; ^3^Houston Food Bank, Houston, TX, United States

**Keywords:** food insecurity, social determinants of health, care coordination, qualitative analysis, systems-level change

## Abstract

**Background:**

Economic and social hardships have worsened food insecurity, particularly among low income and racial-ethnic minority groups. Given the core goal of the 150+ member Houston Health Equity Collective (HEC) to reduce food insecurity by 5% in 2025, we explored member organizations' capacity and challenges faced in screening and responding to food insecurity through care coordination efforts.

**Methods:**

A twice-administered Qualtrics XM survey (Provo, Utah) with 76 organizations, followed by five focus groups with 22 of these organizations, explored reach and response efforts to food insecurity. Qualitative assessments lasted between 0.5 to 1.5 h, were audio-recorded, cleaned, coded, and thematically analyzed using NVivo, version 11 (Burlington, Massachusetts). The qualitative study was guided by a general inductive approach. In total, over 6 h of audiovisual recording were extracted, and over 100 pages of text exported to NVivo for data analysis. The research team read and coded transcripts independently using the codebook, and met routinely to discuss and resolve codes -resulting in numerous revisions to the codebook. Coding structure was discussed at multiple meetings and differences were addressed through consensus. Predominant qualitative themes impacting food insecurity screening were “stigma and cultural-related barriers”, “clinic capacity and attitudes”, “need to focus on upstream influences of food insecurity and SDOH needs”, “impact of COVID-19”, and “need for HEC system responses”. Main recommendations to enhance screening and reach included improving staff culture, enhancing cultural sensitivity across organizational practices, and using shared technology to coordinate care. Respondents stated that the HEC can drive these recommendations through networking opportunities, use of shared resource directory, and placing focus on upstream factors.

**Conclusions:**

Recommendations to target food insecurity must focus on organizational staff responsiveness and sensitivity to patients' needs. Of equal importance is the need for increased attention to the upstream influencers and integration of systems-level interventions to holistically target the barriers impacting food insecurity.

## Introduction

Food insecurity refers to the limited availability and ability to access affordable, nutritiously adequate foods, and is an adverse social determinant of health (SDOH) that impacts approximately 11.1% of U.S. residents and 16% of the Greater Houston area (1, 2). At present, this growing concern is disproportionately higher in low-income families with children and in immigrants, older adults, and communities of color (2). Many deeply intertwined factors impact the occurrence of food insecurity; these include among many, transportation access, geographical isolation, housing instability, employment insecurity, and physical disabilities (3–6). In addition, public health disasters such as the COVID-19 pandemic have amplified the food insecurity burden by destabilizing the food supply chain, disrupting employment, and obstructing consistent access to nutritious foods (7, 8).

Long-term food insecurity over an individual's life course impairs quality of life and is associated with increased risk of chronic conditions such as diabetes and hypertension (9, 10). Research indicates a bidirectional relationship between food insecurity and chronic diseases. It is not uncommon for food insecure individuals to shift limited financial resources from food to other necessary living expenses. When this happens, the likelihood is greater for vulnerable persons to engage in inconsistent eating patterns and overconsumption of energy dense foods that are associated with increased chronic disease risk (11–13). Moreover, those living with chronic condition(s) often experience problems such as increased economic vulnerability, personal health care costs, and job absenteeism, which further perpetuate the cycle of food insecurity (11–13).

In response to growing concerns around food insecurity, healthcare and social service organizations are improving and/or expanding screening approaches, exploring populations' needs and targeting at-risk groups, and are responding more rapidly to food insecurity and co-occurring social needs through increased community engagement (14–18). However, these organizations' responses are often siloed and therefore limited in reach and potential. Thus, transdisciplinary partnerships are timely to advance connections between disparate systems and to optimize care coordination efforts in targeted communities of need (19–21). In response to these complexities around food insecurity, the Health Equity Collective (HEC), a systems-level coalition in the Greater Houston area, was formed in 2018 to reduce food insecurity and other SDOH needs in our region via the development and implementation of a data-driven human-centered ecosystem of care coordination (22). The Health Equity Collective's membership consists of more than 180 multisectoral organizations from healthcare, social services, research, academic, and technology backgrounds. Reducing food insecurity by 5% in 2025 is one of HEC's priority goals, and to that end, operationalizes its operationalizes this work via its backbone team, steering committee, and eight workgroups, of which, includes the food security workgroup (22).

As first steps in HEC's efforts to comprehend the landscape of food security efforts across the Greater Houston area, the food security workgroup qualitatively explored member organizations' capacity, efforts, and processes related to food insecurity screening, referral, and resource coordination in vulnerable populations. A better understanding of these endemic barriers to access and availability within food systems can help us advance systems-level perspectives and strategies for optimizing food insecurity screening and care coordination practices.

## Methods

In August 2020, the food security workgroup distributed its first survey using Qualtrics XM, version August 2021 (Provo, Utah) to HEC members to explore which organizations were currently screening and addressing food insecurity concerns, including intentions to screen for food insecurity in the upcoming year. Organizations were also asked about any follow-up actions in response to patients' testing positive for food insecurity. Following the initial distribution, the workgroup sent out the survey again in October 2020 to increase the response rate. Post second disbursement, seventy-six organizations had completed the survey, which took approximately 30 min to complete. Of these organizations, 39 were not only screening for food insecurity, but engaged in referral and care coordination processes as well. We subsequently summarized descriptive statistics for these organizations addressing food insecurity, using frequency (%) for all categorical variables and mean and standard deviations (SD) for continuous variables. All analyses were conducted using STATA software, version 16.1 (College Station, TX).

After the quantitative survey, we obtained approval from our institutional review board to follow up with organizations that were actively engaged in food insecurity screening and care coordination processes in order to better grasp the various organizational influencers that impeded or enhanced their ongoing work. Following approval, the workgroup held multiple strategy sessions in Fall 2020 to discuss upcoming qualitative assessments. Strategy sessions finalized (i) email invitation scripts for organizations, (ii) qualitative interview script, (iii) data collection method (focus groups), (iv) focus group plan for facilitators and notetakers, and (v) projected timeline for focus groups, data management, and analysis. During these strategy sessions, we finalized the primary goal for the qualitative assessments, and in March 2021, conducted a round of virtual focus group training sessions for eight HEC members who were interested in serving as facilitators and/or note takers. We successfully scheduled and completed five focus groups via WebEx, version 42.8.6.8 (Austin, Texas) with 22 organizations in April 2021 and May 2021, using a developed interview script and guide (Table 1). We conducted all focus groups in English. Each focus group was conducted by a pair of facilitators who successfully completed 2 h of training on how to conduct virtual focus groups. These trainings were conducted by lead researcher and fellow (JCJ) who had amassed extensive formal training in qualitative research as part of her postdoctoral fellowship.

**Table 1:** Focus groups' interview guide.

1. Organizational structure: We are interested in learning more about you and your organization. a. What is your role in addressing food insecurity in your organization? *(Probe: For example, do your screen patients/clients for food insecurity, do you train staff on how to screen and make referrals, do you receive and act on the referrals received, etc.)* b. Can you describe the process of how food insecurity screening (including administering the screening and documentation) occurs within your organization? c. Can you describe the follow-up process after a patient screens positive for food insecurity*?* *(Probe: Can you discuss any use of care coordination platforms to direct patients to community resources?)*
2. These next questions are about screening methods:
a. What staff roles are responsible for screening for food insecurity? b. For those of you with multiple screening staff, can you describe any challenges or successes with this approach? c. If staff training is provided, can you describe the training process? d. Outside the topics that were described earlier, are there any additional challenges/successes you'd like to mention as they relate to the screening process?
3. Many organizations offer some type of resource to address food insecurity: e.g. food pantry navigation, SNAP and WIC enrollment. Can you describe the resources that your organization provides to your target population?
4. These next questions are about population reach and scalability:
a. Can you discuss the patient or client population that is currently being screened? b. Can you discuss your organization's efforts to expand food insecurity screening to a greater catchment area? c. Can you describe whether you currently, or intend to partner with organizations to amplify your food security efforts?
5. How has COVID-19 affected your organization's ability to screen for food insecurity?
*Probe: Were there any factors that either increased the urgency or impeded your current screening process?*
6. How can the Collective help facilitate your approach to addressing food security goals?
*Probe: Can you describe how the availability of a platform with a warm handoff or resource directory might be helpful?*
7. What else on food insecurity screening and care would you like to share with us?

During the focus groups, the facilitators introduced themselves and their varying roles within the HEC; facilitators represented different organizational sectors including academia, healthcare, and community-based. After introductions, facilitators proceeded with verbal instructions and reminded participants that partaking was completely voluntary. In addition to the trained facilitators, one trained notetaker was also present to quietly observe and take notes of the conversations. This was done to safeguard conversations in the event that we experienced difficulties in transcribing our audiovisual recordings. In total, we collected 6 h of focus group recordings and over one-hundred pages of text data, which were transcribed with the support of research interns. We approached data saturation in our fourth focus group, having at that point, interviewed 17 participants. This number tracks with existence research indicating that at 12 interviews, new information is seldom discoverable and that information acquired at such intervals are sufficient to draw insights and reach necessary conclusions (23, 24). Text transcriptions were generated through WebEx. To ensure transcription accuracy, interns compared and verified the auto-generated transcribed texts with the audiovisual recordings. Post focus groups, the facilitators and notetaker debriefed with the lead researcher (JCJ) to discuss the overall flow, observations, and key data takeaways from the interviews.

This study utilized a general inductive approach to frame its qualitative design (25–28). Participants' responses to food insecurity screening and care coordination (including challenges, facilitators, descriptions around settings and targeted population) were the units of analysis. We utilized this qualitative approach for its ease in use, straightforwardness, and systematic application of processes that allowed for the transformation of raw text to discernable themes within the constraints of clear evaluation questions- *as was our case*. Moreover, compared to more restrictive parameters set by other qualitative approaches, this approach allows for the methodological flexibility of thematic analyses (25–28).

Our data management team consisted of a primary (JCJ) and secondary (JG) coder. Text data were exported to NVivo, version 11 (Burlington, Massachusetts), where data were formatted for compatibility. A preliminary codebook, informed by the interview questionnaire, was generated for code extraction by the primary coder. Following this, all digitally-stored transcripts in NVivo were independently read and coded by the coding team. Though guided by the codebook, the coders allowed for data codes to freely emerge from the text resulting in numerous revisions to the codebook. This iterative process of coding and recoding was jointly discussed in routinely convened meetings. Where unanimous agreement could not be achieved, an additional researcher from the HEC was brought on to help resolve differences. After being satisfied as a team with our coding development, codes were finally organized into categories and then categories into broader themes. These themes were subsequently presented to the larger HEC body at workgroup meetings and the HEC quarterly session.

## Results

Thirty-nine (51.3%) of the 76 organizations screened for food insecurity. As shown in Table 2, approximately 56% of these organizations were healthcare sector-related. Approximately 72.4% of organizations relied on the Hunger Vital Sign screening tool (i) “We worried whether our food would run out before we got money to buy more” and (ii) “The food we bought just didn't last and we didn't have money to get more.” (29). Fifty percent of screenings were conducted electronically by staff. Additionally, of these 39 organizations, 60% of organizations were screening partially (meaning, at select sites/locations, but not all) and lastly, only 37.5% of respondents screened for food insecurity at every visit.

**Table 2:** Descriptive statistics on capacity to screen for food insecurity (*N* = 39).

**Variables**	***N***	**Percent**
**Organization sector**	
Healthcare	22	56.4%
Social services	17	43.6%
**Screening tools** ^**a**^		
Hunger Vital Sign screener	21	72.4%
PREPARE tool	3	10.3%
NCCN Distress thermometer	1	3.4%
Other (*Social work assessments, EHR tool)*	4	13.8%
**Screener administration format** ^**b**^		
Electronic self-report	3	10.7%
Electronic staff	14	50.0%
Paper self-report	2	7.1%
Other/Hybrid (electronic and paper)	9	3.2%
**Screening implementation** ^**c**^		
Systemwide	12	40.0%
Partial	18	60.0%
**Extent of population screened** ^**d**^		
0–25%	7	21.9%
26–50%	3	9.4%
51–75%	10	31.3%
76–100%	8	25%
Unsure	4	12.5%
**Screening frequency** ^**e**^		
Every visit	9	37.5%
Quarterly	3	12.5%
Other (monthly, pre-post program attendance, inconsistent)	12	50.0%

^#^missing values: ^**a**^=10, ^**b**^=11, ^**c**^=9, ^**d**^= 7, ^**e**^ =15.

Of the 39 organizations, 22 unique representatives decided to participate in follow-up focus groups, for a response rate of 56.4%. In Table 3, we highlight the demographic breakdown of these 22 individuals who participated in the focus groups on behalf of their organizations. Of the five focus groups, three were female only, one was a mixed group, and the last consisted of male only participants. Across our qualitative sample, 72.7% participants were female, 54.5% were non-Hispanic White, 9.1% were Black/African American, 18.2% were Hispanic, and 18.2% were Asian. Participants represented various sectors: 59.1% were from healthcare, 27.3% were from social services organizations, and 13.6% represented higher-education institutions. Approximately 41% reported screening for other SDOH needs, and 45% said that their organization used technology platforms to help coordinate patients to relevant food assistant programs.

**Table 3:** Descriptive statistics on participants in qualitative focus groups (*N* = 22).

**Variables**	***N***	**Percent**
**Gender**		
Male	6	27.3%
Female	16	72.7%
**Racial/ethnic makeup**		
Non-Hispanic White	12	54.5%
Black/African American	2	9.1%
Hispanic	4	18.2%
Asian	4	18.2%
**Sector representation**		
Healthcare	13	59.1%
Social services	6	27.3%
Higher education	3	13.6%
**Used care coordination platform**		
Yes	10	45.5%
No	12	54.5%
**Screened for other social needs**		
Yes	9	41.0%
No or not mentioned	13	59.0%

### Themes

Table 4 presents our coding structure such that codes are organized into categories and then categories into broader themes. Quotes from these representative themes are presented in greater detail in Table 5. Participants' descriptions of factors impacting food insecurity screening and care coordination efforts largely fell into either challenge or facilitator groupings. Within each theme, we present key emergent barriers or facilitators impacting screening and care coordination efforts.

**Table 4:** Building qualitative narrative from code to categories to theme.

**Codes**	**Categories**	**Themes**
**Patients barred from access due to “undocumented status”**	**Immigration status concerns**	**Stigma and cultural factors either impede or enhances screening/referral efforts**
**Hesitation to disclose status (mistrust)**		
**Screening tool not in native tongue**	**Absence of attention to language and cultural sensitivity negatively impact screening efforts**	
**Absence of clinic translators**		
**Staff not reflective of community**		
**Stigma in *discomfort disclosing verbally to staff***		
**Bilingual staff assist with screening**	**Staff diversity and multicultural support mechanisms enhance screening efforts**	
**Partners serve diverse populations**		
**Use of tailored multicultural approach to screening**		
Staff-volume (*busyness*) reduces quality of time with clients	Clinic capacity challenges impede screening efforts	Clinic capacity and attitudes either deter or improves screening and referral efforts
Technology support among staff needs improvement		
Stigma: the internalized bias based on beliefs, impacts treatment of patients		
More funding needed for referral of resources		
High staff turnover warrants repeated trainings	Poor clinic staff morale impedes screening efforts	
Leadership support and buy in is needed from top down		
Demoralized staff		
Routine staff training and education exists across clinics	Positive staff values on screening enhances response to food insecurity	
Staff is onboard with screening and referral mission		
Partners encounter Issues in accessing EMR	Care coordination issues hinder screening work	
Mistrust in referral “handoff” process		
Need to streamline multiple care coordination platforms		
Not investing in resource directory		
Explore community factors impacting food insecurity	A Systems-level approach is vital to food insecurity reduction efforts	Greater attention to upstream influences of food insecurity and SDOH needs
Food insecurity is part of a larger health care perspective (wellness, disease prevention, etc.)		
Must screen in tandem with other SDOH needs		
Increase collaborative action to drive policy changes		
Explore its ecological entirety: connection to access, cultural sensitivity, poverty, etc.		
Health equity research needed to explore food insecurity	The importance of health equity research to address SDOH needs	
Suspension of in-home social-worker visits	Decline in screening and available resources despite increased food insecurity burden	COVID-19 pandemic impeded reach *or* caused organizations to re-strategize outreach efforts
Suspension of clinical site operations		
Suspension of grocery store tours		
Realignment of financial resources from social to clinical services		
Staff shortages; “Leaner staff”		
Funding sources for food insecurity shut down		
Provision of curbside services to adapt with the times	Retooling of efforts to address food insecurity during pandemic	
“Internal teams would mail out boxes of food”		
Partnering with organizations to expand research		
Facilitate shared learnings and diverse opinions:	Facilitate resource sharing opportunities	Health Equity Collective support in driving recommendations for food insecurity reduction
Provide clinic-level resources for connecting patients		
Provide networking opportunities: “*we do everything through connections”*		
Break down silos- shared grant funding opportunities		
Share/publish evidence on how food security improves health to generate buy-in		
Providing data infrastructure for care coordination platform/ facilitate CIE build	Drive care coordination work	
Provide a “Real resource directory”		
Encourage sharing of research data/collaborative work		
Providing patient-support resources to enhance screening efforts “SNAP application assistance”		
Explore patient/client perspectives on screening experiences	Support and integrate community voice in addressing food insecurity	
Engage and exchange findings with the community: “go out into the community”		

**Table 5:** Representative quotes from each theme in group one.

**Themes**	**Quotes**
Cultural factors either impede or enhance screening/referral efforts	***Q1:** “A lot- because of their documentation status- didn't get any government assistance. Many are day laborers or self-employed with side jobs. They're not eligible for SNAP, and with COVID, the need is really pressing since our patient load in clinic is getting higher.” GG*
	***Q2:** “The success has been that the partners now how to have the staff that speak multiple languages. So definitely with Houston Foodbank, Brighter Bites, Legacy, Hope clinic, - they have multiple people who speak multiple languages. So, that help with the screening for us as a district.” NB*
Clinic capacity and attitudes impacting screening efforts	***Q3:** “If the clinic becomes very busy, they may ask the question to the patient instead of giving it to the patient in the paper format and that becomes challenging because we know that in the verbal format, the patients really don't express it. They're like- yeah no. And the volume of the clinic: It does get missed if there's a lot of people waiting.” EG*
	***Q4:** “With our specific program- we offer direct training to the case workers on how to walk through the tool, they have it in front of them when they are able to screen clients. Most of them kind of end up internalizing it and knowing the questions and know how to guide the conversation without having to read it verbatim. But they do have that as a resource*.” *JK*
Greater attention to upstream influences of food insecurity and SDOH needs	***Q5:** “Food insecurity needs a systems' approach– but an encompassing and encouraging language that's very much a partnership. When you talk to people, they are doing the absolute best they can with what they have– It's not a poverty thing. It's sometimes geographic location or cultural or education level.” TVO*
	***Q6:** “I would like to echo what LH was saying about upstream factors. If we don't fix the bigger system, it doesn't matter what we ask about food- if we don't make a change to the system overall, then people are still going to go to the food pantry and still going to be hungry and still not have access to nutritious food, so I think it's a bigger question or a bigger issue than just food insecurity.” EF*
The COVID-19 pandemic impact on food insecurity	***Q7:** “COVID certainly has been a big challenge, just with loss of access to being in person in some sites…So that's definitely been a challenge. And I think we've also seen a pretty large increase in disconnected phone numbers with COVID, which can make it hard.” LH*
	***Q8:** “We've seen an increase in clientele demand than what we've ever seen, specifically during COVID. With our resource partner network, we've been able to fulfill and meet the demand, but there have been some hiccups in terms of coordination logistics at some points in time.” MH*
Health Equity Collective support in addressing food insecurity reduction	***Q9:** “I found that that what is helpful is like, broadcasting this information to everyone so that we know what other people are doing and can pick up some good ideas from other people and share our ideas. So we're not reinventing the wheel every single time.” SM*
	***Q10:** “The community information exchange infrastructure creates a network and web of integrated systems, [and]. is going to be helpful. Just the sharing infrastructure around it. The CIE from my perspective, feels like the right direction, which does not involve a lot of duplication, but a lot of connection, which is where hopefully, we have systems created in place that can show the impact.” PJ*

### Stigma and cultural factors impacting screening efforts

Participants repeatedly recounted examples of within-clinic or organizational practices that impacted patients or clients' attitudes to screening and seeking post-screening care. In particular, they described how the absence of multicultural support mechanisms - helpful tools to better understand, support, and resolve patients' social needs- were significant challenges that impeded patients' ability to fully communicate their food insecurity needs. Some of these examples included screening tools not being in patients or clients' preferred language, absence of staff translators, and insufficient staff to reflect the community's demographic makeup. Moreover, respondents also described how patients who were undocumented often hesitated to disclose their food insecurity needs as a result of mistrust in staff or fear of reprisal. This underscored the broader issue of bias and perceptions of stigma tied to how they thought they would be treated as food insecurity is often associated with negative societal stereotypes [Quote 1 *(Q1)* in Table 4]. In recognizing some of these challenges, we found that organizations had active measures in place to reach out to their diverse populations, including use of bilingual staff to assist with screening or working with care coordination partners that were accustomed to serving a diverse clientele *(Q2)*.

### Clinic capacity and attitudes impacting screening efforts

Clinic capacity was a common challenge that impacted screening. On this theme, respondents frequently reported how difficult it was to have the type of quality time that they would need with patients to get them to comfortably disclose their food security needs. Getting to that point warrants time and necessary investments in staff-patient relationships, which was reportedly difficult to achieve due to low staff to high patient volume, high staff turnover, staff disinterest or demoralization (at the height of COVID-19), and the lack of buy-in on the importance of screening for SDOH needs *(Q3)*. These examples focus on staff needs and attitudes; yet, there were other challenges such as the need for resources to improve how clinics responded to screening efforts, the need for a technology platform to streamline screening and resource coordination, and the need for more funding and staff trainings to improve efficiency in service delivery *(Q4)*.

### Need to focus on “upstream” influences of food insecurity and SDOH needs

Participants also believed that to improve organizational culture around screening and care-coordination, the current ecosystem has to evolve in adopting a more integrative systems-level approach to intervening on food insecurity. Participants who responded to this theme stated that food insecurity was part of the wider health and wellness ecosystem, and that to comprehensively address it, various sectors must think of food insecurity beyond a poverty lens. Furthermore, while it is important to screen for food insecurity in tandem with other social needs as part of a holistic approach to needs resolution, institutions must equally recognize that other factors including geographical or cultural limitations are key determinants of food insecurity as well *(Q5)*. According to participants who responded to this theme, addressing food insecurity via a systems-level lens will require fixing the system from its current state *(Q6)*. This will require a shifted focus from that of largely screening, to one that welcomes collaborative actions in driving policy change, and the inclusion of community perspectives better understand the community driven factors that impede and enhance progress to food insecurity resolution.

### COVID-19 pandemic impeded reach and caused organizations to re-strategize outreach

The impact of COVID-19 was a predominant theme across all five focus groups. It was clear that the pandemic inflicted significant blows to organizations that addressed food insecurity as a programmatic focus. As a direct consequence of the pandemic, many organizations experienced declines in screening rates as well as reduced ability to provide resources to their target population. Despite an increase of food insecurity experiences across their service populations, organizations were unable to have these needs fully met due to staff shortages, depleted funds, redirection of financial resources away from SDOH needs to clinical services, and suspension of on-site services and interactions with target populations *(Q7)*. However, despite these challenges, some organizations reported adapting existing procedures to retain patient/client engagement, including providing contactless delivery options and leveraging partnerships to maintain or expand outreach *(Q8)*.

### The need for system responses facilitated by the HEC

This theme was neither a challenge nor facilitator. Rather, it spoke to the broader need for coalitions to be engaged in systems-level intervening of food insecurity and other social needs. Through participants' responses, we found that the HEC was strongly positioned to provide its broad network of multisectoral partners with resource sharing and networking opportunities, including avenues to share learnings and best practices, so that organizations do not have to constantly “reinvent the wheel” in addressing SDOH needs *(Q9)*. Furthermore, participants believed that the HEC could further drive system change by providing the required data infrastructure to (i) enable organizations to pursue collaborative grant funding mechanisms and (ii) build and house a resource directory and technology platform that optimizes care coordination practices *(Q10)*.

## Discussion

The purpose of this study was to explore organizational efforts in addressing food insecurity with the goal that findings and recommendations be leveraged to improve screening processes, inform care coordination process, and reduce food insecurity prevalence over time. The focus groups revealed various factors that either impeded or helped facilitate screening and referral of needed resources. We found that challenges and facilitators were rooted in system-wide themes of social environment, physical environmental, organizational, and policy-related influences. This emergent ecological perspective provided a wider understanding of the many complex and interrelated factors that amplify food insecurity and related social needs within our city's most vulnerable populations. The completion of the five focus groups revealed five distinct salient themes that organizations can act on to enhance screening and dissemination efforts. These narratives create a clearer roadmap for HEC on how to facilitate, leverage, build, and link organizations (partnerships) to enhance food security efforts across the region.

On the theme that stigma and cultural-related factors (cultural sensitivity, multicultural mechanisms) either impeded or enhanced screening/referral efforts, we found that the organizations that failed to account for their population's cultural needs in screening protocols faced significant setbacks in getting patients to disclose food insecurity. As Bernhardt and King discuss, patients are often hesitant to talk about being food insecure for fear of being judged or seen as “less than” due to the economic and social hardships they experience; and to add, the apparent lack of clinic cultural support mechanisms (screening tools in preferred language, confidential screening with interpreter services, diverse clinic providers, etc.) further compound communication challenges between patient and clinic staff (30). With the U.S. minority population projected to exceed 50% by 2050, integration of actionable, cultural-adapted solutions into the current healthcare model is very much needed to mitigate access-to-care barriers, which unsurprisingly, disproportionately impact racial-ethnic populations. Many organizations have already adapted or are pursuing recommendations from existing scientific literature and toolkits to enhance screening and address the needs of their diverse populations. Some of these strategies include (i) employing, training, and retaining diverse and multilingual staff to effectively communicate with patients/clients, (ii) mandating staff cultural competency trainings to better address patient-perceived stigmas and staff biases that may emerge during the patient care experience, (iii) fostering a culture of diversity and inclusion in organizational values and leadership, (iv) recruiting and retaining resource coordination partners that understand diverse clientele' needs and can provide resources that align with their cultural preferences, and (v) provide health education and promotion material in culturally-appropriate communication tools to diverse clientele (31–36).

Another theme impacting screening was clinic capacity and staff attitudes. Additionally, in part, owing to the COVID-19 pandemic, organizations experienced high staff turnover and low staff morale, the result of which were inevitable disruptions in workflows and institutional best practices. As is the case with frequent staff turnover, organizations must train and bring new employees up to speed on organizational culture and procedures to effectively address patients' needs. It is highly probable that there will also be an ongoing need for cultural competency trainings as new employees navigate best approaches to adequately address diverse patients' needs (30). In response, a leadership structure that recognizes the clinical and public health significance of SDOH screening and timely interventions to both preserve patients' health and lower heath care utilization costs is strongly recommended (17, 37, 38). To that end, encouraging organizations to integrate SDOH interventions as part of their broader focus on health equity will require trainings on screening practices and proficiency in technology usage and care coordination to meet the needs of this region's rapidly growing diverse population (16, 17, 39, 40).

Third, organizational participants also wanted an increased focus on “upstream” solutions in research, health, and social service interventions. Presently, resolution efforts in food insecurity largely center on acute resource coordination and delivery. These strategies help provide immediate relief to vulnerable populations. However, these efforts are not permanent solutions, nor do they adequately account for the complex structural and social environment interactions that surround and impact overall health and social wellbeing (41). For example, many focus group participants highlighted how food insecurity exists alongside adverse social needs such as transportation, housing, and economic vulnerabilities. To that end, targeted interventions must recognize the embeddedness of individuals within systems, and in response, integrate more comprehensive socio-ecological (interpersonal, institutional, community, and policy) approaches that sustain long-lasting impact on behavior change, health outcomes, and health equity (42–44). Notwithstanding issues stemming from competing organizational priorities and sharing sensitive patient data, institutions stand to benefit from enhanced productivity when they willingly (i) leverage collaborative opportunities for data exchange, grant funding, and multi-level program delivery approaches, (ii) jointly participate in developing resource directories to facilitate closed-loop care coordination efforts, and (iii) link silos of excellence in SDOH efforts to create holistic ecosystems of care that address co-occurring SDOH needs at multiple levels of influence (45–47). These actionable approaches have been strategically operationalized across the HEC's workgroup sessions, networking events, and ongoing efforts to build an integrative technology platform that coordinates care delivery between healthcare and social services sectors.

Lastly, the impact of COVID-19 emerged as a predominant barrier to screening and coordination efforts. Many of these organizations, having been supported by grant funding and volunteer work, discussed how COVID-19 impacted financial and staffing capabilities to address food insecurity and other social needs. Respondents spoke of both suspending screening and referral efforts as staff and funding priorities shifted from SDOH needs to COVID-related acute care, and of the financial limitations tied to caring for an increasingly food insecure population. Similarly, nationwide and regional reports have projected increases in the national food insecurity rate, with disproportionate burden on households with children and in racial-ethnic minority groups (8). There is much to learn from organizations who regrouped and retooled their efforts to address these challenges (14, 15, 48). In particular, South Carolina's Office of Rural Health and First Nations Health Authority documented their own experiences, and available toolkits serve as a collective blueprint for those seeking direction on how to adequately prepare for service delivery during public health emergencies (14, 15). Recommendations such as curbside distribution of resources to minimize patient contact, telehealth education and service delivery, and building partnerships with sister-organizations to expand reach and service delivery are some of the many considered approaches for addressing food access during public health crises.

As the themes and representative quotes show, food insecurity is an equity issue that disproportionately impacts communities of color and the economically vulnerable. Its tentacles are rooted in complexed and often intertwined social and environmental challenges including poverty, geography, migrant status, access and eligibility, and health outcomes. Immediate resolution may provide brief relief, but fails to capture the ecosystem of up-stream factors that influence prolonged need for ongoing food assistance. From our experiences as a growing coalition and in examining organizations' responses, the HEC plays a critical intervening role in some of the major impediments to screening and resource coordination. The HEC's existing shared impact strategy helps provide the framework for creating a level playing field for organizations to learn best strategies to circumvent these existing barriers. Moreover, the HEC will continue to actively (i) share evidence-based learnings and networking opportunities, (ii) provide data infrastructure for care coordination, and equally important, and (iii.) build the shared community platform that integrates the voices of community members in planning and decision-making processes to equitably target health needs.

Of equal importance, it should be the goal of coalition work -exercised through coalition members efforts- to gradually steer the current landscape from one that addresses food insecurity through short-term, immediate assistance to one that embraces an impact-driven systems-level perspective to problem solving (48). This intentional transitioning will require the integration of key organizational players at the intersection of community-voice, advocacy, healthcare, and community social services to (i) capture threats to food security within the ecosystem, and (ii) engage policy experts and the wider system to develop and ultimately adopt culturally-sensitive policies that minimize inequities around access, cost, and eligibility. It is expected that the level of policy adoption (organizational, local, state, federal) will vary across systems, as different regions adopt relevant policies to meet the gaps and needs of their vulnerable populations. However, clear insights from the Greater New Haven Coordinated Food Assistance Network (CFAN) demonstrate universal examples of how food systems can adopt processes that have far-reaching impact. This includes collaborations between organizations and local city government to support summer meal programs, elevating the roles of resident leaders in municipal decision-making processes to provide insights on lived experiences, and elevating the voices of those who are under-represented to help formulate and implement actionable strategies that provide sustained relief to those facing food insecurity (48).

Despite this study's many strengths, there were multiple limitations, which could profoundly impact the transferability of our findings. This study was a convenience sampling of participants, which reduces transferability of results to the wider public. Secondly, we conducted focus groups virtually. It is possible that participants were more likely to be disconnected and less engaged. Similarly, it was very difficult to observe participants' body language, which impeded our ability to add context to responses. Still, given that most participants worked virtually and had varying work and travel schedules, the virtual setting presented a strong advantage for getting participants together who otherwise, would not have been able to meet. Other strengths of the study must also be noted. The ability to explore responses from those engaged in screenings and care coordination is a huge step forward in helping to normalize processes that center “culturally sensitive” screening and response strategies across the care continuum. Furthermore, rallying multiple organizations' perspectives on these issues will significantly enhance screening efforts, care coordination response, and health equity work on food insecurity and related social needs across the region and nation by extension.

## Conclusion

In this coalition-driven study to explore HEC members' efforts in addressing food insecurity across the Greater Houston region, we relied heavily on qualitative assessments to better understand the challenges that impact screening and care coordination and the possible strategies that may reduce food insecurity through improvements in screening and care coordination practices. Clinic capacity and staff attitudes, impact of COVID-19, stigma and cultural-related barriers, and lack of upstream attention to SDOH needs were obstacles to food insecurity screening and resource coordination efforts. However, themes such as improved staff culture, cultural sensitivity, and use of shared technology to coordinate care, can play a salient role in improving organizational response to food insecurity. To that end, organizational response to food insecurity may be best served by optimizing organizational staff responsiveness and sensitivity to patients' needs, as well as equal leveraging of collaborations through coalition-building to drive systems-level change and reduction efforts across vulnerable populations.

## Data availability statement

The raw data supporting the conclusions of this article will be made available by the authors, without undue reservation.

## Ethics statement

The studies involving human participants were reviewed and approved by the University of Texas Health Science Center (UTHealth) Committee for the Protection of Human Subjects. The Ethics Committee waived the requirement of written informed consent for participation.

## Author contributions

JJ and JG: conceptualized the project, conducted the analysis, and wrote the original draft of the manuscript. SGC, HM, JA, EG, and NB: helped in reviewing and editing the manuscript. SS: aided in conceptualization, project administration, writing and editing, and supervision of project. All authors contributed to the article and approved the submitted version.
